# Efficacy and safety of low-power holmium laser enucleation of the prostate: experience gained from more than 700 cases

**DOI:** 10.55730/1300-0144.6075

**Published:** 2025-08-17

**Authors:** Bahadır TOPUZ, Can SİCİMLİ, Ahmet HALİS, Engin KAYA, Sercan YILMAZ, Murat ZOR, Serdar YALÇIN, Selahattin BEDİR

**Affiliations:** 1 Urohealth Urology Clinic, İstanbul, Turkiye; 2 Department of Urology, Elmadağ Dr Hulusi Alataş State Hospital, Ankara, Turkiye; 3 Department of Urology, University of Health Sciences, Yedikule Chest Diseases and Thoracic Surgery Hospital, İstanbul, Turkiye; 4 Department of Urology, Liv Hospital Ulus, İstanbul, Turkiye; 5 Department of Urology, Acıbadem Bodrum Hospital, Muğla, Turkiye; 6 Department of Urology, University of Health Sciences, Gülhane Training and Research Hospital, Ankara, Turkiye

**Keywords:** Benign prostatic enlargement, enucleation, holmium laser, HoLEP, low-power HoLEP

## Abstract

**Background/aim:**

The aim of this study was to investigate the functional outcomes, safety, and effectiveness of low-power (LP) holmium laser enucleation of the prostate (HoLEP) using a 37.5 W holmium laser source.

**Materials and methods:**

We retrospectively reviewed 713 patients with a diagnosis of benign prostatic hyperplasia (BPH) treated with HoLEP using an LP setting (37.5 W laser set to 1.5 J with a frequency of 25 Hz). All procedures were performed by experienced urologists. Functional outcomes, perioperative parameters, and complications were assessed over a 12-month follow-up period. Effect sizes (Cohen’s d) and 95% confidence intervals (CI) were calculated to evaluate the magnitude and precision of changes in maximum urinary flow rate (Qmax), postvoiding residual volume (PVR), international prostate symptoms score (I-PSS), and other parameters.

**Results:**

The mean (SD) preoperative serum PSA level was 4.8 ng/mL (3.6), and the mean prostate volume was 94.02 mL (55.35). The average enucleation time was 72.42 min (32.36), morcellation time was 10.35 min (8.62), and total operation time was 82.74 min (38.08). At 1 month after the surgery, significant improvements were observed in I-PSS (from 25.01 (7.62) to 11.02 (5.3), Cohen’s d = 2.08, 95% CI 1.91–2.25), Qmax (from 10.7 (4.28) to 18.68 (9.52) mL/s, d = 1.08, 95% CI 0.93–1.22), and PVR (from 139.38 (18.07) to 24.85 (18.07) mL, d = 3.13, 95% CI 2.87–3.39), all with p < 0.001. Transfusion was required in 23 patients (3.2%) and early reoperation in 14 patients (2%). Stress urinary incontinence occurred in 11 patients (1.5%) during late follow-up but was resolved in all cases by the 6th month. These outcomes support the safety and efficacy of LP HoLEP, yielding results comparable to those reported with high-power (HP) laser techniques in the literature.

**Conclusions:**

In this retrospective study, we showed that LP HoLEP can be performed safely and effectively, similar to other enucleation methods; however, further studies are necessary to validate its acceptance as a viable alternative to HP HoLEP.

## 1. Introduction

Benign prostatic hyperplasia (BPH) is a common disease in older men. Significant advances have been made in the treatment of BPH in recent years [1]. Prostate enucleation surgery using laser energy is gaining popularity as a treatment, and is fast becoming the gold standard treatment for BPH [2]. Compared to standard transurethral resection of the prostate (TURP), holmium laser enucleation of the prostate (HoLEP) offers better hemostasis, shorter catheterization and hospitalization times, and nullifies the risk of TURP syndrome while improving voiding symptoms.

The technique and results of HoLEP were published for the first time by Gilling et al. [3] in 1996. Since then, the development of laser technology, enucleation and continence preserving techniques, and endoscopic instruments with lower calibration have reduced the complication rates of this surgery [4]. The ability to perform surgery regardless of prostate volume provides convenience for both the patient and the surgeon in the postoperative period [5]. HoLEP is among the options in the surgical treatment of BPH in all current guidelines [6].

HoLEP is generally implemented at a high-power (HP) of 80–100 W. The HP setting of HoLEP has the advantage of high enucleation efficiency because it can remove prostatic adenomas within a short time. On the contrary, the high cost of HP lasers prevents the wider use of HoLEP. Currently, HP lasers are standard for HoLEP, and it is believed that low-power (LP) lasers are not as effective for this procedure. LP devices (20, 30, and 50 W) are available in nearly all operation rooms with lower set up costs and simpler requirements. Rassweiler et al. [7] was the first to show that LP settings (24–39.6 W) are effective, suggesting potential cost savings. However, LP HoLEP has not gained much support amidst the rise of HP machines.

This study examined the effectiveness and safety of a 37.5 W holmium laser for HoLEP using a 3-lobe enucleation technique to explore its potential for broader adoption.

## 2. Materials and Methods

### 2.1. Study population

The data of patients who underwent LP HoLEP surgery between January 2017 and December 2022 in a tertiary center were retrospectively reviewed. The study included patients with a maximum urinary flow rate (Qmax) below 15 mL/s, an international prostate symptom score (I-PSS) over 12, and those who had not responded to medical treatment for benign prostatic obstruction (BPO). Patients were excluded if they had a history of urethral or prostate surgery, prostate cancer, urethral strictures, or neurogenic bladder diagnosed through urodynamic testing. No prospective follow-up protocol was implemented in this study. Patients with incomplete retrospective data were excluded from the analysis.

Demographic data of the patients, preoperative prostate volume measurements, preoperative serum prostate-specific antigen (PSA) levels, and hemoglobin (Hb) levels were recorded. Perioperatively, enucleation time, morcellation time, total operation time, laser energy level, and resected tissue weight were also recorded. Postoperatively, the duration of the hospital stay, catheter duration, stress urinary incontinence (SUI) or urge urinary incontinence rates, transfusion requirement, clot retention, presence of prolonged hematuria, presence of urinary tract infection, bladder neck stenosis, urethral stenosis, and the need for reoperation were evaluated. The effectiveness of the operation was evaluated by performing uroflowmetry postoperatively in the 1st, 6th, and 12th month after the surgery.

### 2.2. Methods

In this retrospective study, all procedures were performed by 5 urologists in our center. A single pedal holmium laser (VersaPulse, Lumenis Inc., Santa Clara, CA, USA) with enucleation and hemostasis power of 37.5 W (1.5 J × 25 Hz) and a 550 nm end-firing fiber (Slim-Line 550, Lumenis Inc.) were used for prostate enucleation. A 26-French continuous flow resectoscope (Karl Storz, Tubingen, Germany), a rigid nephroscope with a 5 mm working channel (Karl Storz, Germany), and a tissue morcellator (Lumenis Inc.) were utilized during surgery. The 3-lobe technique was used with early apical release to perform the enucleation, as previously described [4]. The patients were operated on in the lithotomy position under general or spinal anesthesia. All patients had a 3-way 22-French catheter inserted with bladder irrigation at the end of the procedure. Since the patients were operated on by 5 different surgeons, the patients were divided into 2 groups (the first 500 cases and the other 213 cases) based on the number of patients for which each surgeon completed their learning curve.

Ethics committee approval was received from the Gülhane Scientific Research Ethics Committee of the University of Health Sciences on 22 November 2022 with decision number 2022-347. Written informed consent was obtained from all patients.

### 2.3. Statistical analyses

All statistical analyses were performed using SPSS version 23.0 (IBM, Armonk, NY, USA). Descriptive statistics were reported as mean (SD) for continuous variables and as frequency and percentage for categorical variables. The normality of distributions was assessed using the Kolmogorov–Smirnov test. For comparisons of continuous variables between more than 2 groups, one-way analysis of variance (ANOVA) was used followed by Tukey’s honestly significant difference (HSD) posthoc test. For categorical variables, the chi-square test or Fisher’s exact test was applied, as appropriate.

Where applicable, effect sizes (Cohen’s d) and 95% confidence intervals (CI) were calculated to provide a better understanding of the magnitude and precision of the differences observed. A p-value of less than 0.05 was considered statistically significant. As this was a retrospective study, patients with missing or incomplete data were excluded from the final analysis.

## 3. Results

### 3.1. Patient characteristics

This retrospective study included 713 patients with a mean (SD) age of 62.43 years (7.34). The mean preoperative serum PSA level was 4.8 ng/mL (3.6) and the mean preoperative prostate volume was 94.02 mL (55.35). The baseline characteristics of the patients are detailed in Table 1.

### 3.2. Perioperative data

The perioperative data is summarized in Table 2. The average enucleation time was 72.42 min (32.36) (first 500 cases was 76.42 min and last 213 cases was 50.05 min), the average morcellation time was 10.35 min (8.62), the average total operation time was 82.74 min (38.08), the average laser total energy was 94.55 J (42.19), and the average total resected tissue weight was 52.56 g (39.7).

### 3.3. Postoperative data

Table 3 presents the functional parameters at baseline and follow-up, and Table 4 represents postoperative complications regarding enucleation surgery. Statistically significant improvements in I-PSS, quality of life (QoL), Qmax, average urinary flow rate (Qave), and postvoiding residual volume (PVR) were observed 1 month postsurgery compared to preoperative levels, and these improvements continued over time (p < 0.05). At the 12-month follow-up, statistically significant improvements were noted in Qmax (10.7 mL/s (4.28) at baseline vs. 24.84 mL/s (8.5) at 12 months), PVR (139.38 mL (18.07) vs. 15.48 mL (23.75)), I-PSS (25.01 (7.62) vs. 6.75 (3.04)), and QoL (5.03 (0.94) vs. 1.27 (1.01)) (p < 0.05).

The average postoperative hospital stay was 24 h (3.88), and the average catheter duration was 32.17 h (5.89). In the early postoperative period, 23 patients needed a transfusion, 23 patients had clot retention, 12 patients needed recatheterization, 50 patients had hematuria, 13 patients had urinary tract infections, and 14 patients had to be reoperated. In the late postoperative period, SUI was seen in 11 patients, bladder neck contracture in 14 patients, urethral stenosis in 11 patients, postmicturition inability in 12 patients, and postmicturition dribbling in 22 patients. SUI was resolved in all patients at the 6th month follow-up.

## 4. Discussion

Over the past 2 decades, HoLEP has become a widely recognized, minimally invasive treatment option for BPH regardless of prostate size, offering an alternative to TURP and open prostatectomy (OP) [1]. Although the early adopters of HoLEP suggest using HP lasers (≥80 W) for the procedure [3], there is limited data available on the use of LP HoLEP [7–11]. LP devices (20, 30, 50 W) are available in nearly all operation rooms with lower set up costs and simpler requirements. However, more data regarding the effectiveness and safety of LP HoLEP in enucleation surgery is required before wider adoption. Compared to other studies on this topic, our study uses one the largest patient cohorts reported to date.

Rassweiler et al. [7] conducted a retrospective analysis of patients undergoing HoLEP using power settings of 25 W (n = 39) and 40 W (n = 45). The enucleation efficiencies for these groups were 0.45 g/min and 0.71 g/min, respectively. In our study enucleation efficiency was 0.68 g/min (0.29) for the first 500 cases and 1.05 g/min (0.48) for the last 213 cases. The two groups differed by the level of experience of the surgeons. The first group of 500 cases represented our first experience with LoLEP and the enucleation efficiencies were similar to those reported in the literature, which are between 0.5 and 1.1 g/min [8] (Table 5). Consistent with previous findings, the enucleation efficiencies improved for the last 2013 cases, as the surgeons had gained more experience [8]. The reported enucleation efficiency with HP lasers (100 W or 120 W) reaches up to 1.48 g/min [9]. Our results showed an average enucleation efficiency of 1.05 g/min, showing that acceptable outcomes can be obtained with a 37.5 W laser.

Another important criteria for LP laser surgery is the enucleation time. In a previous study, the mean enucleation time was 75.5 min with a 20 W laser and 54 min with a 37.5 W laser [7]. Another study reported a mean enucleation time of 45.4 min with a 30 W holmium laser [9]. In our study, enucleation was performed with a 37.5 W laser and the mean enucleation time for the first 500 cases was longer than reported in other studies, at 76.42 min. However, after the first 500 cases, it decreased to 50.05 min, more in line with other studies. According to the literature, the learning curve for HoLEP is more challenging compared to other endoscopic BPH surgeries. The average learning curve is estimated to range between 20 and 80 cases. Accordingly, during the first 500 cases in our study, 5 urologists each performed approximately 100 procedures. A marked reduction in enucleation time and a significant improvement in enucleation efficiency were observed in the subsequent 213 case. These findings can be attributed to the surgeons having completed their learning curves.

In our study, we conducted the LP HoLEP procedure following the method described by Tunç et al. [5]. In this technique, the early release of the sphincter and mucosal flap, which is a landmark of sphincter preservation, ensures a safe, effective, and fast enucleation. Once the correct enucleation plane is identified during HoLEP, the outcome depends on the technique more than on the power of the laser. The 12-month follow-up data of LP HoLEP in our study showed statistically significant relief of obstructive symptoms, corroborated by I-PSS assessment, in patients with lower urinary tract symptoms (LUTS) caused by symptomatic BPO (p < 0.05). Improvements in voiding parameters (Qmax and PVR) and symptom scores (I-PSS and QoL) after LP HoLEP at 12 months were comparable to those observed with HP and LP HoLEP [8]. Additionally, the median PSA reduction of 79.17% at 6 months post-LP HoLEP indicates complete removal of the prostatic adenoma, aligning with results from previous HP HoLEP studies [8]. This supports our findings that LP HoLEP can be effectively carried out using a 37.5 W holmium laser.

Regarding immediate complications, we recorded 63 patients with Clavien grade 1 (50 with hematuria) and grade 2 (13 with urinary tract infection) complications, and 26 patients with Clavien grade 3a (12 with recatheterization) and grade 3b (14 with reoperation) complications. No Clavien grade 4 or 5 complications were reported.

When enucleation has been performed with an LP (25 W or 40 W) holmium laser, the blood transfusion rate has varied from 0 to 8% [7,8,11]. A randomized controlled study showed that there was no statistical difference between LP and HP lasers on blood transfusion rate [12]. In our study, the blood transfusion rate was 3.2%. While this is comparable with other studies, it can be reduced by more surgical experience. The rate of immediate postoperative reinterventions (Clavien 3a/3b) in our study aligns well with findings from randomized HP HoLEP studies and metaanalyses [8].

The most concerning complication of HoLEP surgery is SUI. The incidence of SUI following HoLEP has been reported in several studies as 4.9–12.5% [13,14]. In our study, the rate of SUI was 1.5%, which is lower compared to the literature. This rate decreased to zero by the 6-month follow-up. We believe that this low incidence is the result of the surgical technique used and the fact that the functional results are so well documented.

As the length of hospital stays increase, so does the hospital-acquired infection rate, increasing the burden on the healthcare system [15]. In our study, the average hospital stay was 24 h (3.88). This short hospital stay is a distinct advantage for patients and healthcare professionals over conventional endoscopic methods and open surgery.

One of the most important difficulties with surgical BPH treatments for patients and healthcare professionals is the need to reoperate. In various studies, reoperation rates for transurethral interventions have been reported between 13 and 14.7% for open surgery, and around 8% for endoscopic surgeries [16,17]. The reoperation rate after HoLEP surgery was 4.2% in a single center, retrospective study [18]. In our study, reoperation was required in 14 patients (2%). These cases coincided with the learning curve time. This result shows that by performing anatomical enucleation, sufficient tissue can be removed and the need for reoperation will decrease.

Kim et al. [19] compared robot-assisted simple prostatectomy (RASP) and HoLEP as surgical treatment options for BPH. The duration of catheterization and hospital stay were notably shorter in patients who underwent HoLEP compared to those who received RASP. Furthermore, the postoperative decrease in hemoglobin levels was significantly less pronounced in the HoLEP group. The rate of transient SUI was significantly lower in the RASP group at the 2-month follow-up. Similarly, Umari et al. [20] found transient SUI to be significantly less common in the RASP group compared to the HoLEP group (8.9% vs. 1.2%, p = 0.035). Although the reported incidence of transient SUI after HoLEP generally ranges between 3 and 5% in the literature, some studies have reported higher rates. In our study, the rate of transient SUI was 1.5%, which is relatively low.

Several metaanalyses comparing LP and HP laser HoLEP have reported that LP laser systems are associated with reduced enucleation efficiency relative to HP systems [21,22]. Nevertheless, the rates of postoperative complications and functional outcomes were found to be comparable between the two approaches. These findings are consistent with the results obtained in our study.

In our study, the incidence of urethral stricture was 1.5%, while bladder neck contracture was observed in 2% of patients. These findings are comparable to the results reported by Glienke et al. [23], who evaluated long-term outcomes, characteristics, and risk factors of strictures following holmium laser enucleation of the prostate (HoLEP). In their study, the overall rate of urethral stricture was 5%, and bladder neck contracture was reported in 2% of cases. These rates were influenced by factors such as surgical technique, surgeon experience, and patient-specific characteristics. The relatively low complication rates observed in our cohort may be attributed to meticulous apical dissection, careful morcellation, and the use of LP laser settings. Our results support the notion that LP HoLEP is associated with a low incidence of postoperative strictures, in line with the long-term findings of Glienke et al. [23].

## 5. Conclusion

Despite its benefits, HoLEP has not been widely adopted globally compared to TURP. This is partly due to its steep learning curve and the high initial cost of the laser equipment. The HP holmium laser, which is nearly 3 times more expensive than the LP holmium laser, requires special infrastructure like high-current sockets and advanced air-conditioning systems, adding to the overall cost. However, studies have shown that HoLEP can be cost-effective in the long term, especially when considering the lasting relief from LUTS and the shorter hospital stays compared to TURP and OP. Our study is one of the largest in the literature, by using data from more than 700 patients. However, the main limitations of the study are its retrospective nature and lack of randomization. The results showed that LP HoLEP has high enucleation efficiency, minimal perioperative complications, and excellent functional outcomes at the 12-month follow-up. Our findings emphasize that the skill in performing the HoLEP technique is more critical than the power of the laser alone. While LP HoLEP can be as safe and effective as well-established enucleation techniques, further randomized, prospective studies are necessary to validate its acceptance as a viable alternative to HP HoLEP.

## Figures and Tables

**Table 1:** Patient characteristics and preoperative data.

Parameters	Mean ± SD
Age (years)	62.43 ± 7.34
PSA (ng/mL)	4.8 ± 3.6
Preoperative Hb (g/dL)	14.28 ± 1.35
Postoperative Hb (g/dL)	13.85 ± 1.44
Prostate volume on TRUS (mL)	94.02 ± 55.35
Parameters	n (%)
Preoperative retention/catheter groups	32 (4.4)

PSA, prostate-specific antigen; Hb, hemoglobin; TRUS, transrectal ultrasonography.

**Table 2:** Perioperative data.

Parameters	Mean ± SD
Enucleation time in the first 500 cases (min) a	76.42 ± 32.36
Enucleation time in the last 213 cases (min) a	50.05 ± 14.64
Enucleation efficiency in the first 500 cases (g/min) b	0.68 ± 0.29
Enucleation efficiency in the last 213 cases (g/min) b	1.05 ± 0.48
Morcellation time (min)	10.35 ± 8.62
Morcellation efficiency (g/min)	6.91 ± 3.18
Total operation time (min) d	82.74 ± 38.08
Total laser energy (j)	94.55 ± 42.19
Laser efficiency (j/g)	2.26 ± 0.98
Resected tissue weight (g)	52.56 ± 39.7

a Measured from insertion of the laser fiber until removal,

b resected weight/enucleation time,

c resected weight/morcellation time,

d measured from insertion until removal of the resectoscope.

**Table 3:** Functional outcomes at baseline and follow-ups.

Parameters	Follow-up				
	Baseline Mean ± SD	1st month Mean ± SD	6th month Mean ± SD	12th month Mean ± SD	p a
Qmax	10.7 ± 4.28	18.68 ± 9.52	22.78 ± 9.23	24.84 ± 8.5	0.006
Qave	8.1 ± 3.3	10.7 ± 4.5	11.8 ± 3.7	11.9 ± 3.4	0.001
PSA level (ng/mL)	4.8 ± 3.6	1.9 ± 0.7	1.6 ± 0.6	1.4 ± 0.7	0.001
I-PSS	25.01 ± 7.62	11.02 ± 5.3	9.05 ± 1.4	6.75 ± 3.04	0.001
QoL	5.03 ± 0.94	1.6 ± 1.01	1.32 ± 0.97	1.27 ± 1.01	0.001
PVR (mL)	139.38 ± 18.07	24.85 ± 18.07	19.88 ± 21.75	15.48 ± 23.75	0.001

I-PSS, international prostate symptom score; QoL, quality of life; Qmax, maximum urinary flow rate; Qave, average urinary flow rate; PVR, postvoiding residual volume.

a Statistically analyzed with one-way ANOVA and Tukey HSD test.

**Table 4:** Postoperative data.

Parameters	Mean ± SD
Hospitalization time (h)	24 ± 3.88
Catheter time (h)	32.17 ± 5.89
Parameters	n (%)
Transient stress urinary incontinence	11 (1.5)
Transfusion	23 (3.2)
Clot retention	23 (3.2)
Recatheterisation	12 (1.7)
Hematuria	50 (7.1)
Urinary tract infection	13 (1.8)
Bladder neck contracture	14 (2)
Urethral stricture	11 (1.5)
Reoperation	14 (2)
Postmicturition inability	12 (17)
Postmicturition dribbling	22 (3.1)

**Table 5:** Enucleation efficiency in selected patient cohorts [8].

Reference	Enucleation efficiency a (gm/min)
Becker et al.	1.11
El-Hakim et al.	0.34
Seki et al.	0.50
Montorsi et al.	0.93
Elzayet et al.	0.63
Petersen et al.	0.83
Matlaga et al.	1.48
Current study (first 500 cases)	0.68
Current study (last 213 cases)	1.05

a Resected weight/enucleation time.

Table revised from the table of the publication by Becker et al. [8].
